# Probiotics and Trained Immunity

**DOI:** 10.3390/biom11101402

**Published:** 2021-09-24

**Authors:** Naima G. Cortes-Perez, Alejandra de Moreno de LeBlanc, Jorge G. Gomez-Gutierrez, Jean Guy LeBlanc, Luis G. Bermúdez-Humarán

**Affiliations:** 1 Université Paris-Saclay, INRAE, AgroParisTech, UMR 0496, 78350 Jouy-en-Josas, France; 2 Centro de Referencia para Lactobacilos (CERELA-CONICET), San Miguel de Tucuman, Tucuman T4000ILC, Argentina; demoreno@cerela.org.ar (A.d.M.d.L.); leblanc@cerela.org.ar (J.G.L.); 3 Department of Child Health, School of Medicine, University of Missouri, Columbia, MO 65201, USA; jgdk2@health.missouri.edu; 4 Micalis Institute, Université Paris-Saclay, INRAE, AgroParisTech, 78350 Jouy-en-Josas, France

**Keywords:** probiotics, trained immunity, immune response, human health

## Abstract

The characteristics of innate immunity have recently been investigated in depth in several research articles, and original findings suggest that innate immunity also has a memory capacity, which has been named “trained immunity”. This notion has revolutionized our knowledge of the innate immune response. Thus, stimulation of trained immunity represents a therapeutic alternative that is worth exploring. In this context, probiotics, live microorganisms which when administered in adequate amounts confer a health benefit on the host, represent attractive candidates for the stimulation of trained immunity; however, although numerous studies have documented the beneficial proprieties of these microorganisms, their mechanisms of action are not yet fully understood. In this review, we propose to explore the putative connection between probiotics and stimulation of trained immunity.

## 1. Introduction

Innate immune defenses include hematopoietic (myeloid and lymphoid) and non-hematopoietic cells and involves cell-dependent and cell-independent mechanisms. The first includes phagocytosis and cytotoxicity, whereas the second involves the action of secreted factors such as antimicrobial peptides, cytokines, alarmins, chitinases, proteases, etc. [1]. Earlier, it was believed that the innate immune response was deprived of memory; however, in 2011, Netea et al. showed that innate immunity exhibits a primitive mode of memory called “trained immunity” [2]. This new perception has modernized our understanding of the immune response and encouraged researchers to further study how this primitive memory works. The ability of a host to protect itself against potentially harmful agents is the oldest natural act of preservation, present in all living organisms from prokaryotes to eukaryotes. In this context, recent works have shown that archaea and bacteria have a mechanism of protection similar to an adaptive immune system [3]. These defense systems are mediated by some genetic elements named CRISPR (clustered regularly interspaced short palindromic repeats) in association with *cas* genes, to create the CRISPR-Cas system [4,5]. In this system, three phases can be distinguished: (i) uptake of foreign genetic elements to build a memory library, (ii) production of CRISPR RNA (crRNA) from collection, and (iii) target blocking [4]. Thus, CRISPR could represent an example of a primitive immune memory, based primarily on the rearrangement of genetic elements. In the same line, Reimer-Michalski and Conrath [6] compared trained immunity with immune-memory developed by plants. Plants, unlike mammals, do not develop an adaptive immune response; however, they are able to remember infections. The mechanisms used by plants to enhance the immune response involve epigenetic reprogramming of genes in order to organize cells to respond to subsequent infections [6]. Epigenetic reprogramming is understood to be the hereditary process that modifies the gene expression activity without altering the sequence of the gene (e.g., DNA methylation and Histone modifications) [7,8].

These data support the idea that trained immunity is a process used by primitive organisms devoid of an adaptive immune response, and that it has been adapted and maintained in vertebrates, as proposed by Netea et al. [2,9]. These authors also described trained immunity as a functional reprogramming experience by innate immune cells after contact with an insult, to remember the interaction and prepare the cells to respond to other, non-specific challenges [10,11]. Some experiments carried out in mice showed that interaction with certain microbial components could protect them from infection with a different microorganism (i.e., a non-specific protection). Even if the mechanisms of trained immunity are not yet fully understood, recent observations suggest that epigenetic reprogramming associated with metabolic modifications play a major role [2,11,12]. In this context, the best-characterized examples of trained immunity are those produced by the BCG (Bacille Calmette-Guérin) vaccine [2] and the β-glucans of *Candida albicans* [12].

The purpose of this review is to explore the putative connection between probiotics and the stimulation of this primitive immune response. Therefore, in this article, we will review epigenetic mechanisms, ligands, and receptors, as well as immune cells linked to the reprogramming of trained immunity. We will focus on the documented mechanisms of action of probiotic bacteria that could be associated with the stimulation of trained immunity.

## 2. Epigenetic Mechanisms Potentially Involved in Trained Immunity

### 2.1. Epigenetic Regulation

**Figure 1:**
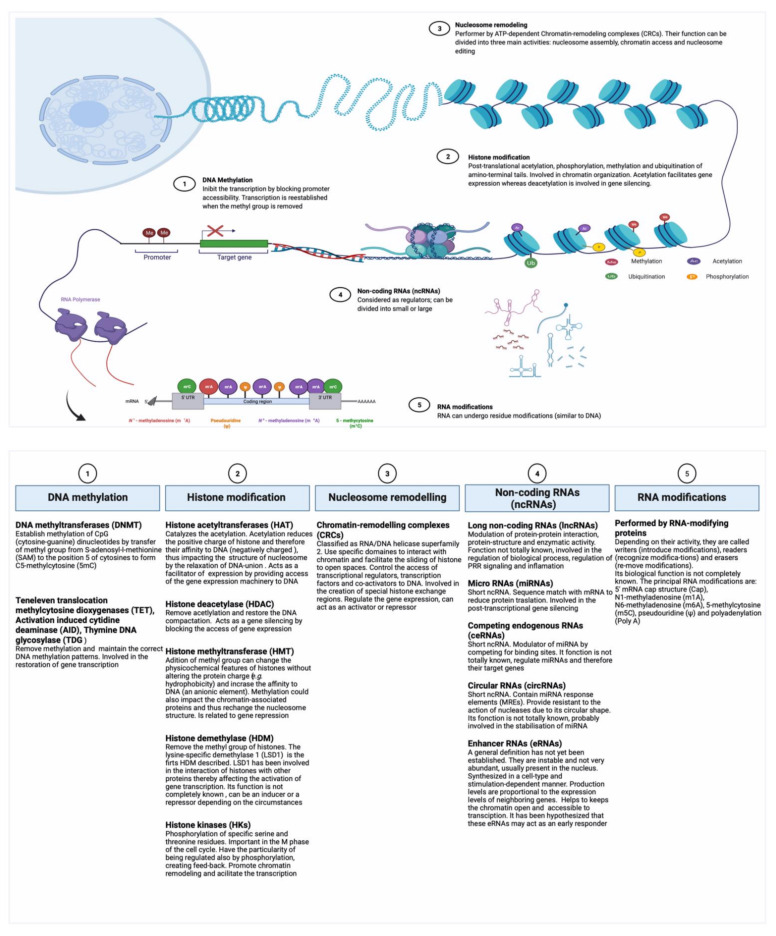
Epigenetic mechanisms involved in trained immunity. Adapted from “Cancer Epigenetics, Icon Pack–Epigenetics, Epigenetics and Gene Expression and Regulation of Transcription in Eukaryotic Cells” templates (Biorender). Retrieved from https://app.biorender.com/biorender-templates, accessed on 23 September 2021). For more information see refs. [13,21,22,23,24,25,26,27,28,29,30,31,32,33,34,35,36,37].

An epigenetic change is a superficial modification made in the genome that does not involve alterations in the DNA sequence. Currently, five main types of epigenetic regulation can be distinguished: (i) the methylation of DNA, conducted by the action of DNA methyltransferases (DNAMT) (ii) the post-translational modifications of histones, performed by the Histone acetyltransferase (HAT), (iii) the nucleosome remodeling by the action of macromolecular complexes ATP-dependent, (iv) the family of non-coding RNAs (ncRNAs), and (v) the RNA modifications (e.g., methylation) in protein-coding and ncRNAs (also known as epitranscriptome) [13,14,15,16]. Figure 1 compiles information regarding these types of epigenetic regulations, their effectors molecules, and some identified properties. The epigenetics mechanisms involved in trained immunity are not yet fully understood; however, the best-described are those induced by β-glucan and BCG. An example of this was described by Kleinnijenhuis et al. in 2012 [17]. Indeed, these authors showed that BCG use NOD2 receptor and the action of histone 3 lysine 4 trimethylation (H3K4me3) to induce epigenetic changes at the level of histone. In this context, DiNardo et al. (2021) elegantly described the current evidence of beneficial and/or detrimental epigenetic modifications performed by innate cells in the process of trained immunity [18]. The general process has been perfectly summarized by Khader et al. [19]; trained immunity starts with the recognition of signals through cellular receptors (e.g., NOD2 for BCG and Dectin 1 for β-glucan); however, other intermediate signals intervene to induce the epigenetic changes. These intermediate signals are not fully elucidated, but have been linked to metabolic changes, especially in relation to glycolysis. Moreover, other studies showed that the accumulation of some substrates of the Krebs cycle (e.g., succinate and fumarate) can inhibit the activity of demethylases which directly impacts on the histone activity [19]. However, further studies are needed to better understand the mechanisms. In a recent review by Koeken et al. 2021, the authors describe how the new omics-based technologies (e.g., epigenomics, proteomics, metabolomics etc.) can be used to facilitate the study of trained immunity by allowing its analyses at different levels of activity [20].

### 2.2. Probiotics as Potential Epigenetic Regulators

In order to understand how indigenous microorganisms and/or their metabolic products interact with the epigenome, Shenderov pointed out the importance of creating an international alliance around the Human Gut Microbiota and Epigenomic Project, complementing existing epigenomics projects by including the study of the role of indigenous microorganisms in these processes [38]. In 2017, Carbonero expressed his surprise at discovering that a search on PubMed for “human epigenetics microbiome” returned only a few records [39]. The same search, carried out four years later, in 2021, resulted in more than 850 records. Nevertheless, it should be noted that a current PubMed search of the two words “epigenetics and probiotics” together reveals only 125 results. Although this represents an increase, the connection between the epigenetic impact of probiotics and the repercussions on the host, for example, on trained immunity, is not fully understood. All this makes it evident that further studies are needed to elucidate the possible mechanisms involved.

Based on the association between microbes and microbial components and cancer, Nackerdien hypothesized that if the active compounds are identified, probiotics could be used in immunotherapy [40]. In an interesting article published in 2010, Licciardi et al. evoked the putative implication of probiotics in the induction of epigenetic modifications and highlight the importance of studying this. In their hypothesis, they state: “*…the biological activity of probiotics are mediated through complex epigenetic changes that regulate the activation status of key transcription factors involved in host immunity*” [41]. This hypothesis was later be expanded with the association of nutritional factors, proposed by Canani et al., who provided interesting data on some nutritional factors, their epigenetic roles, and their potential impact, particularly in early life [42].

The first documented work involving the study of epigenetic consequences of probiotic in humans was performed by Worthley et al. in 2009 [43]. In their work (a randomized double-blind, placebo-controlled study), they used the prebiotic HAMS (high-amylose maize starch) and the probiotic *Bifidobacterium lactis* LAFTI B94 alone or as a combined symbiotic preparation. The researchers analyzed: inflammatory markers in serum, the bacterial profile and the concentration of short-chain fatty (SCFA) in feces, the histology of intestinal crypts, and the DNA methylation of some promoter regions, including some related to colorectal cancer. Their observations showed that administration of HAMS combined with *B. lactis* did not have a major impact on most of the parameters measured, but was linked with a modification of bacterial-profile and with a lowest methylation of the loci *MINT-2* (methylated in tumor-2). However, the authors considered these data non-conclusive due to the absence of other related markers [43]. These observations are consistent with other findings that support that diet (as well as other external factors) can prompt beneficial changes in the microbiota profile, which could be potentially heritable, a concept known as the hologenome theory [44]. In addition, the epigenetic potential of diet, gut microbiota, and microbial components, on the immune response priming and disease susceptibility has been widely discussed [38,45,46]. In this context, Bebek et al. studied the relationship between three factors: (i) microbial profile, (ii) neck squamous cell carcinoma (HNSCC), and (iii) the methylation status of four genes (*MDR1, IL8, RARB,* and *TGFBR2*) linked to HNSCC and inflammation [47]. Their results showed an association of HNSCC with the methylation status of *MDR1* and *IL-8* genes. More importantly, in the context of HNSCC, *MDR1* gene methylation was correlated with two specific bacterial subpopulations (Enterobacteriaceae and Tenericutes), whereas *IL-8* methylation could not be associated with any particular profile [47]. The study of epigenetic modifications addressed by microorganisms was initially focused on pathogenic bacteria, because they were considered mechanisms used to control the host’s immunity. The cascade of actions induced by the lipopolysaccharide (LPS), an agonist of host inflammatory signaling responses, which can come from an external source (infection), internal production (microbiote), or as food contamination, is a well-known example [48,49]. However, the study of the epigenetic modifications performed by lactic acid bacteria (LAB) remains largely unexplored. Nevertheless, in 2012, Ghadimi et al. took into consideration the knowledge about the activity induced by LPS (e.g., stimulation of histone acetylation and expression of inflammatory cytokines) in inflammatory bowel disease (IBD) and decided to study the epigenetic impact of LAB using an in vitro LPS-induced model (a 3D co-culture model composed of human intestinal HT-29/B6 or T84 cells and PBMCs that mimics the interaction of bacteria with immune cells via the intestinal cells) [50]. The study included two probiotic bacteria (*Bifidobacterium breve* DSMZ 20,213 and *Lactobacillus rhamnosus* GG (LGG)) and was centered on the impact of these strains on IL-23 and IL-17 expression (two cytokines involved in IBD) and the underlying induction pathways. They found a reduction of IL-23 and IL-17 cytokines (lymph nodes, confirming the anti-inflammatory activity of that *B. breve* and LGG). These observations were linked to the reduction of histone acetylation and NF-kB activity, concomitant with an increase level of DNA methylation [50]. Thus, based on the ability of probiotic bacteria (and/or their metabolites) to favorably modulate different immune and metabolic pathways, other researchers were interested in the study of the probiotics as a dietary supplement with a focus on the therapeutic potential of these microorganisms, especially regarding cancer [51], diabetes [52], and allergy-related diseases [53]. However, in spite of this, the specifics elements used by probiotics to interact with the host remains poorly understood. In this context, Bhat and Kapila, in 2017 [54], conducted a review on the epigenetic impact of metabolites derived from gut microbiota and some probiotic strains. Among the modifications described by the authors, there are two metabolites (butyrate and acetate) that induce inhibition of histone deacetylases and histones phosphorylation and acetylation, respectively. They also cite some probiotic bacteria that affect DNA methylation (such as *Lactobacillus acidophilus*, *Bifidobacterium breve*, and LGG) and regulate histone deacetylases, such as *Akkermansia muciniphila* [54]. Additionally, recent evidence presented by Lenoir et al. demonstrated the impact of the commensal bacterium *Faecalibacterium prausnitzii* in the regulation of *Dact3*, an epigenetic regulator of Wnt/beta-catenin signaling [55].

In the past, immunologists were mainly focused on the protection against potentially dangerous agents. This could explain why the study of the immune response induced by beneficial agents remained in the background for several years. Although a change is currently taking place to separate *protective* from *pathogenic* immune response, most of the current scientific knowledge is related to the immunological response against pathogens.

## 3. Signal Detection and Immune Response

**Figure 2:**
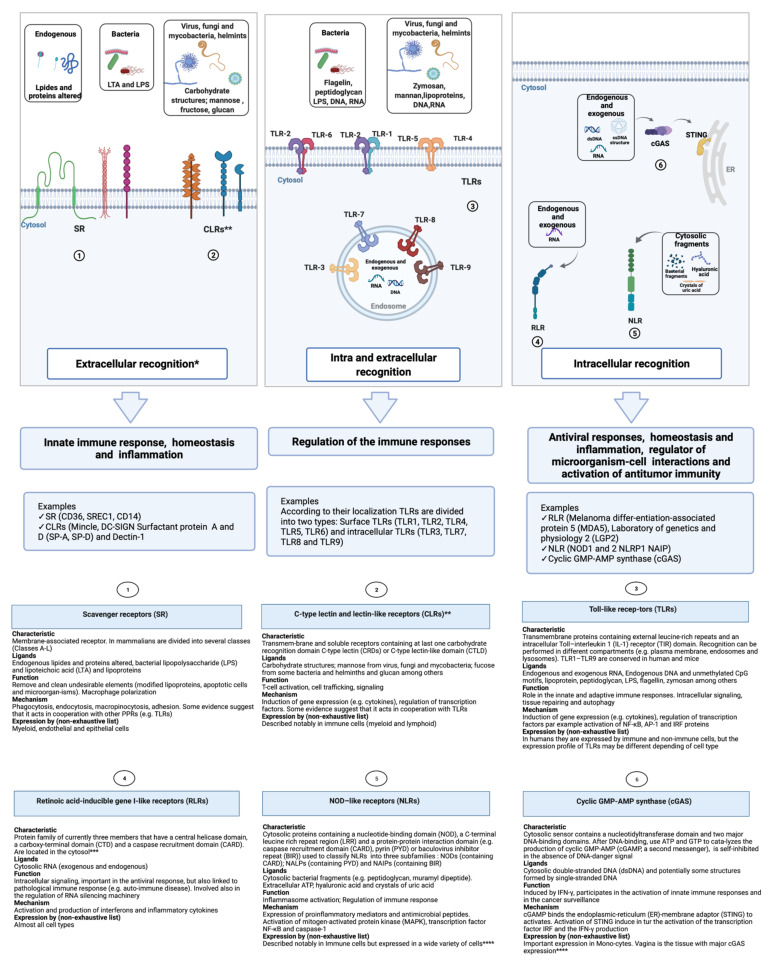
Molecular patters and receptors impacting innate immune response, homeostasis, and inflammation. * Despite the research efforts, there remains Babylonian confusion concerning the nomenclature and classification of Scavenger receptors (SR) and C-type lectin and lectin-like receptors (CLRs). Please note that the classification presented here is of a general nature for the purposes of this review; however, different classifications have been proposed and can be found in the literature. For example, C-type lectin-like receptors (CLRs), which have a scavenger activity, have been considered as Scavenger receptor (SR) class E (SR-E), e.g., Lectin-like oxidized LDL receptor 1 (LOX1) and Dectin-1 (also classified as CLRs) [63,64]. ** C-type lectin receptors bind to carbohydrates in a calcium-dependent manner, whereas C-lectin-like receptors can bind proteins, lipids, and carbohydrates in a calcium-independent manner; the CLRs abbreviation includes the two types [65]. *** Recent evidence suggests that it may also be located in the cell nucleus [66]. **** The expression profile could be different according to cell type and tissue (see Uhlén et al. [67] and the related database “Human Protein Atlas” available online: http://www.proteinatlas.org accessed on June 2021). Adapted from “Detection of PAMPs by Toll-Like Receptors (TLRs) and Innate Immune System: Cellular Locations of Pattern Recognition Receptors “templates (Biorender). Retrieved from https://app.biorender.com/biorender-templates, accessed on 23 September 2021). For more information see refs. [59,63,68,69,70,71,72,73,74,75,76,77,78,79,80,81,82,83,84].

In mammals, the innate immune system comprises physical and chemical defenses and involves hematopoietic (i.e., myeloid and lymphoid) and non-hematopoietic cells (that are linked to other organs or systems), which means that practically all tissues could be involved [1]. The sensing process performed by the immune response is centered on the recognition of conserved molecular patterns, a concept reviewed previously [56]. Pattern recognition receptors (PRR) represent a group of proteins that are able to detect conserved microbial molecules present on the potentially dangerous agents [57]. In this context, Koenderman et al. referred to two different recognition patterns: intra- and extra-cellular sensing [58]. The first one represents a primitive form which triggers a more direct response without the intervention of complex systems. Conversely, the second one is a more sophisticated way of sensing (probably as a result of evolution) present in multicellular organisms, and uses more complex systems involving cellular and non-cellular actors [58]. Figure 2 summarizes the principal pattern recognition receptors (PRRs) including the Toll-like receptors (TLRs), RIG-I-like receptors (RLRs), NOD-like receptors (NLRs), C-type lectin receptors (CLRs), Scavenger receptors (SR), and cyclic GMP-AMP synthase (cGAS) [13,59]. Among the pathogen-associated molecular patterns (PAMPs, also known as microbial-associated molecular patterns or MAMPs) are mannans (a yeast cell wall component) and bacterial cell wall agents such as lipopolysaccharide (LPS), formylated peptides, peptidoglycans, and teichoic acids [60]. It should be noted that self-molecules could also be recognized, as has been long proposed by Matzinger’s danger theory [61], such as the damage-associated molecular patterns (DAMPs, such as heparan sulfate, cytochrome c, and galectins) produced by a tissue damaged by non-microbial causes, and the lifestyle-associated molecular patterns (LAMPs, such as cholesterol crystals, uric acid, prions, and prion-like protein danger signals) [62]. Thus, the recognition of PAMPs/DAMPs/LAMPs by PRRs induces a signaling cascade that allows the activation of transcription factors that in turn regulate the genes involved in the immune response; however, sensing can also have a direct effect at the chromatin level to induce the gene expression.

**Figure 3:**
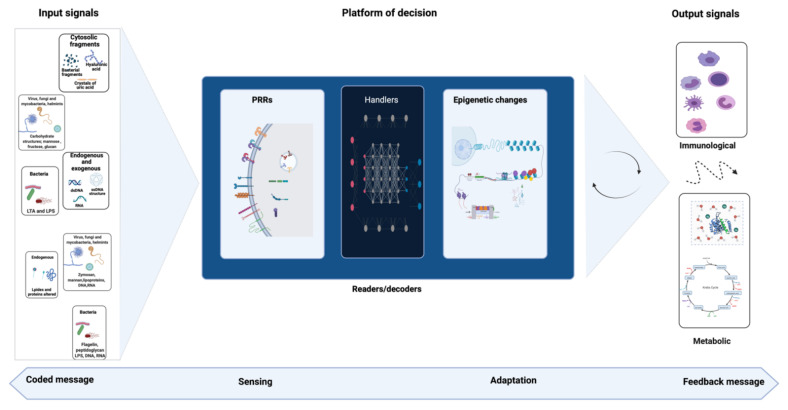
Handler elements connecting sensing and response (this figure was created with Biorender.com, accessed on 2 September 2021).

Accordingly, crosstalk between signals and detectors could be summarized as a basic communication process in which a message is encoded by molecules such as PAMPs/DAMPs. The code is recognized by the receptors of the sensing system (e.g., PRRs) activating a cascade of signals to decode the message and adapt the system to the stimulation received. Thus, it seems evident that to deal with a constant flow of messages, the system must have a very precise decoding interface. Analogous to computer programming, the key elements of this hypothetical interface will be called “handlers” here. Once a stimulus is detected by the receptor, the information is then transferred to handlers, which will search in their memory more information about this signal; the result may be the epigenetic regulations, which will then be used to make a decision and launch the most appropriate feedback process (result of the cascade of signals). More details are included in Figure 3 to better understand this analogy and the mechanism involved. Even if the existence of a decoding interface and its handling elements remains to be proven, the aforementioned adaptation could be translated into an epigenetic reprogramming that allows the readjustment of functional conditions necessary at that time (e.g., expression, regulation, and/or inhibition of molecules), thus impacting the immunometabolism [13].

## 4. Immune Cells Subjected to Trained Immunity

The interplay between immunological and metabolic process is known as immuno-metabolism and has emerged as an important area of study [85]. As expected, the metabolic processes associated with the immune response are strongly connected with the stimulated cell and its function. Therefore, the epithelial cell will have a different metabolic reaction than a hematopoietic cell and the reaction will be different in a macrophage than in a lymphocyte. In this context, O’Neill et al. detailed different, closely linked metabolic pathways that play a key role in immunometabolism, such as glycolysis, Krebs cycle (also named tricarboxylic acid cycle (TCA), pentose phosphate pathway, oxidation (and synthesis) of fatty acid, and amino acid metabolism [85].

**Figure 4:**
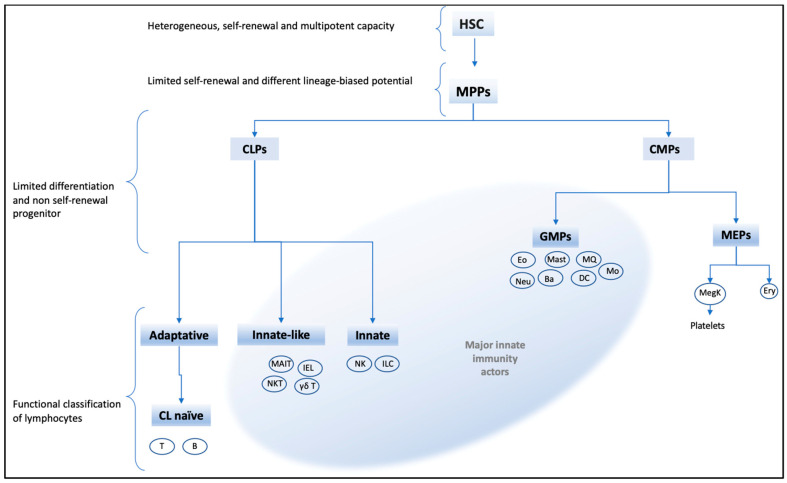
Hematopoietic hierarchy adapted with a functional classification of lymphocytes.

Hematopoiesis is a complex process involving pluripotent and self-renewal heterogenic stem cells (HSC). These HSC are regulated by epigenetic, metabolic, and immunologic stimuli to form different blood cell lineages [86,87]. Recent research supports the existence of several HSC subtypes produced as a result of the level of expression of surface markers and their response to signal pathways [88]. Thus, the study of the HSC gene expression and plasticity concerning HSC subtypes, notably in adulthood, could provide useful insights to better understand how different stimuli can regulate the innate immune response. In this context, the hierarchical organization of hematopoietic cells is still debated and various models have been proposed in adults [89,90,91]. For the purposes of this review, we will use an adaptation of hematopoietic hierarchy and functional classification of the mentioned authors (Figure 4).

**Figure 5:**
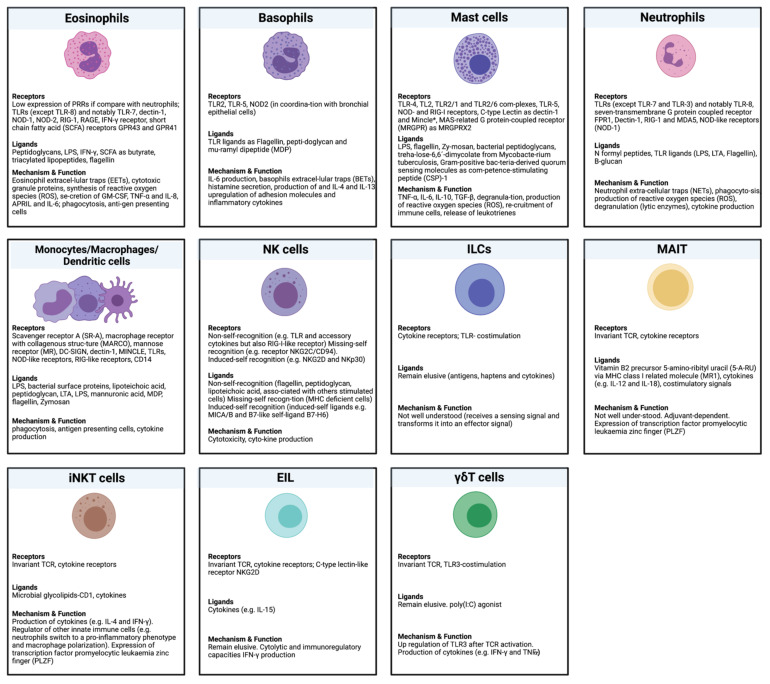
Principal hematopoietic actors in the innate immune response (this figure was created with Biorender.com, accessed on 20 September 2021). For more information see refs. [92,93,94,95,96,97,98,99,100,101,102,103,104,105,106,107,108,109,110,111,112,113,114,115,116,117,118,119,120,121,122,123,124,125,126,127,128].

The major hematopoietic actors of the innate immune response communicate (directly or indirectly) with microbial factors that can modulate them. In Figure 5, some characteristics of mentioned elements are described. Here, we want to emphasize cells that can directly interact with microorganisms and/or their components. Although the expression of TLRs has been detected in some types of lymphocytes, the direct interaction of innate- and innate-like lymphoid cells with microorganisms has not been completely established, and their possible functional activities remain controversial [92,93]. In this context, some information concerning the evidence of innate immunity memory in mice and humans has been reviewed by Netea et al. [10]. Given the complexity of the data and because monocyte-macrophages cells play a major role in the innate immune response, the next section will focus on these cells from granulocyte-macrophage lineage in order to review the hallmarks associated with trained immunity. A list of microbial-macrophage receptors involved in the innate immunity was published by Plüddemann et al. [94]; in the future, this list could be used as a basis to explore the epigenetic regulations associated with the stimulation of these receptors.

Innate immune cells can undergo different immunological adaptations (e.g., *differentiation*, *trained immunity*, *tolerance*, and *priming*), which cannot be easily differentiated when they are studied; as a result, this subject has recently been addressed by some researchers [129,130,131]. To avoid confusion, Divangahi et al. proposed to define the particularities of each one of these functional rearrangements [129]. Accordingly, *differentiation* represents the change from an immature to a mature state; in *priming* (or active transcription), the level of the immune response produced by the first insult does not decrease to basal levels and the second insult is then additional. In contrast, in *trained immunity,* when the stimulus is removed, the immune response returns to its basal state; however, the pre-established epigenetic alterations are maintained and the response to the second stimulus is stronger. Finally, another adaptation completely contradictory to the previous one is the *tolerance*; in this situation, the cell fails to respond to the second insult due to a kind of gene-expression barrier [129]. Thus, both trained immunity and its counterpart, tolerance, can be generated in a non-specific way following PRR-mediated re-stimulation; however, while trained immunity results in a hyperresponsive state, tolerance leads to a hyporesponsive state and, unfortunately, little is known about this paradoxical involvement of PRR by microbial ligands. In this context, Ifrim et al. developed a series of in vitro experiments to better understand how functional reprogramming is selected in monocytes [130]. They found that upon the first interaction with microbial ligands, monocytes are altered in a way that depends on the nature of receptor involved and concentration of stimulatory ligands and receptors. High concentration of NLR ligands result in trained immunity (enhanced production of TNF-α and IL-6); similarly high concentration of TLR ligands leads principally to a tolerance status. However, low concentrations of NLRs ligands had no remarkable effects and low doses of TLR ligands stimulated the immune response [130]. Although the authors showed interesting results, the question of how the reprograming processes can be differentiated remains unanswered because the greatest difficulty in studying innate immunological adaptations resides in the fact that they use the same mechanisms to produce different results. Currently, there is no model able to analyze (in a single system) all the different adaptations executed by the immune cells, which constitutes a scientific challenge. Another interesting study performed by Cheng et al. revealed how glucose metabolism plays a fundamental role in trained monocytes via the dectin1-Akt–mTOR–HIF-1α pathway [132]. Their results also support the idea that a switch from glycolysis to oxidative phosphorylation can move the LPS response towards a tolerance, through an event that involves the activation of the enzyme deacetylase sirtuin-1 (inhibited in trained monocytes) [132]. In this line, Saeed et al. focused on the research of an epigenetic signature that could designate the future functional state (tolerance or trained immunity) on the monocyte. Their observations include the demonstration (in vitro and in vivo) of an important role of cyclic adenosine monophosphate (cAMP) signaling in trained immunity [133]. This result is interesting because it is known that increased cAMP levels in innate immune cells leads to an attenuated immune response [134,135], strongly suggesting a flexible and regulatory role of cAMP that should be explored in detail in the context of innate immune memory. Unfortunately, despite the efforts made in recent years to understand the mechanisms involved in trained immunity and endotoxin tolerance, they have not been fully elucidated [136]. Table 1 lists some of the markers that have been observed in trained immunity.

## 5. Hypothetical Role of Probiotics in Trained Immunity

Probiotics have been defined as: “*live microorganisms which when administered in adequate amounts confer a health benefit on the host*” (World Health Organization, 2001); however, the mechanisms underlying the probiotic action has still not been fully understood. The host’s immune stimulation by probiotics has been reported in many works, including those that use animal models (healthy animals or diseased models) and human clinical trials [143,144,145]; however, only scarce articles link the terms “trained immunity” and probiotic. The work of Torpee et al., about the mechanism exerted by the probiotic *Rhodobacter sphaeroides* SS15 to control acute hepatopancreatic necrosis disease caused by *Vibrio parahaemolyticus* in cultivated white shrimp, represents one of these rare examples [146]. As was explained earlier in this review, the lack of articles describing trained innate immunity as a mechanism associated with probiotics may be due at least in part to the focus of this new concept on pathogenic organisms.

An approximation of mechanisms by which probiotics stimulate innate immune memory can be obtained with a revision of mechanisms exerted by the gut microbiota. Many probiotic microorganisms originate from this niche. In addition, it is known that one of the mechanisms by which probiotics can affect the host’s immune response is indirectly through beneficial modifications of gut microbiota [147]. The potential role of gut microbiota on trained immunity was recently reviewed [148]. The authors explained that microbiota-derived ligands, products, or metabolites are MAMPs that bind PRRs present on innate immune cells, such as monocytes/macrophages and NK cells. This cell activation can induce the epigenetic and metabolic reprogramming necessary for the increased responsiveness observed upon the subsequent pathogenic exposure, which also include the release of certain cytokines. Furthermore, the authors proposed that microbial ligands can travel through the bloodstream and interact with hematopoietic progenitors in the bone marrow to induce long-term memory and enhance myelopoiesis to mount a better and faster response against systemic infections. This non-specific innate immune memory was described as an evolutionary process used by the host to protect itself against different pathogens [10]. However, under certain circumstances, this symbiotic relationship between the host’s immune system and its commensal microbiota may cease to be beneficial and lead to chronic inflammatory disorders, such as autoimmunity, allergies, and metabolic syndromes [149]. Thus, the selective modulation of the intestinal microbiota would have an important therapeutic potential, and probiotics appears as ideal candidates for evaluation, in order to improve the balance between microbiota and the immune system.

As is the case for the gut microbiota, probiotics can exert pro- or anti-inflammatory effects [150]. Probiotics that enter the organism by the oral route could act similarly to the established gut microbiota. They (or their so-called postbiotic products or metabolites) can serve as MAMPs that train innate immune cells to generate enhanced, rapid, and non-specific responses against secondary stimulation, e.g., secondary pathogen’s challenge. Even when the term trained innate immunity was not used, according to the results present in many articles, it can be proposed that this mechanism was associated with the beneficial preventive effect of some probiotics, most of them at the intestinal level. In this sense, Llewellyn and Foey revised the ability of probiotics to modulate innate immune responses through direct or indirect effects on signaling pathways such as NF-κB, MAPK, JAK/STAT, and PI3K/Akt signaling [151]. These authors described the actions of probiotic microorganisms on intestinal epithelial cells (IEC), DCs, neutrophils, and macrophages; moreover, they highlighted that the mechanisms of action of probiotics are specific to each strain [151]. In a mouse model, the oral administration of *Lactobacillus casei* CRL 431 decreased the severity of infection caused by a strain of *Salmonella enterica* serovar Typhimurium (*S. typhimurium*) obtained from the Children’s Hospital in San Miguel de Tucuman (Tucuman, Argentina) [152]. This effect was associated with increased intestinal expression of TLR2, TLR4, and TLR9, and improved production and secretion of certain cytokines (TNF-α, IFN-γ, and IL-10) in Peyer’s patches previous to the infection. In another article, the beneficial effects of probiotic lactobacilli on the innate immune response against rotavirus infection was reviewed [153]. The authors described how certain probiotic strains can enhance the production of type I IFN and IFN-γ in the intestinal mucosa, which was related to the enhanced antiviral response associated with the regulated expression of inflammatory cytokines and chemokines. On the other hand, it has been shown that the probiotics *L. acidophilus* ATCC 53,103 and *B. infantis* ATCC 15,697 have an impact on the regulation of SIGIRR (Single Ig IL-1 Related Receptor), which limits the intestinal inflammation and promote commensal microbial colonization [154,155].

The effect of probiotics and their metabolites or cell components on the hematopoiesis was also described [156]. Although the mechanisms involved are not exactly known, components of the probiotics’ cell wall that would reach the circulation from the intestinal mucosa were associated with this effect. In addition, modifications in the levels of circulating cytokines induced by probiotics may influence the recovery of myelopoiesis. In an immunosuppressed model, selected probiotic strains (*L. casei* CRL431, *L. rhamnosus* CRL1506 or CRL 1505) were able to induce early recovery of myeloid cells in the bone marrow, which was also associated with enhanced recruitment of phagocytic cells to infectious sites and increased resistance against infections [157,158].

It was also reported that probiotic administration can affect the intestinal microbiota and gut-associated immune cells in early life [159]. In a mouse model, the administration of fermented milk containing *L. casei* DN-114001 into the mothers during the suckling period and to their offspring after weaning positively improved the intestinal microbiota and stimulated non-specific immune cells, such as IgA+ cells, macrophages, and DCs. This effect could also be related to the training of certain immune cells that later protected the mice when they were challenged with the same *S. typhimurium* strain used by Castillo et al. (2011), inducing a faster and more effective response against this pathogen in the offspring [160].

Furthermore, the existence of the common mucosal system allows to explains the effect of probiotics administered orally in sites other than the intestine, such as the lungs [161]. The trained immunity stimulated by probiotics may be associated with the faster response against respiratory pathogens observed in the hosts that received these beneficial microorganisms. As an example, non-infected mice fed with *Bifidobacteium longum* MM-2 enhanced IFN-γ production in Peyer’s patches and splenic NK cell activity; moreover, when they were challenged with influenza virus, NK cell activity was significantly enhanced both in the spleen and lungs and the animals showed decreased virus proliferation and suppression of inflammation in lungs with increased expression of NK cell activators genes, such as IFN-γ, IL-2, IL-12, and IL-18 [162]. This protective effect can be associated with the stimulation of alveolar macrophages and/or NK cells activity in the airway mucosa exerted by probiotics [163]. In another article, Koizumi et al. demonstrated that oral administration of mice with *L. pentosus* S-PT84 significantly enhanced splenic NK cells’ activity and induced NK1.1-positive NK and NK T cells to produce IFN-γ. This increase in IFN-γ production was dependent on IL-12 produced by CD11c1 DC after the interaction of the bacteria with the TLR2 and/or TLR4 present on the surface of DCs [164]. It is also important to consider that, in the immune environment of the mesenteric lymphoid nodes (MLN), DCs stimulated by probiotics or their components or metabolites influence the development of T and B cells that migrate to the lungs via the lymphatic system [165].

Numerous studies have shown the pro-inflammatory induction aptitude of several probiotics, notably the production of TNF-α and IL-6; however, this pro-inflammatory profile is dependent on the strain used. This observation, pointed out by several works, is cited in the comparative study performed by Dong et al. [166]. In this sense, one of the best-described models of trained immunity is that exerted by the β-glucans from *Candida albicans*, which involves the dectin-1 receptor on monocyte/macrophage cells [12]. It should be noted that these β-glucans (polysaccharides of D-glucose linked by a β-bond) are not exclusive to *C. albicans*. In fact, β-glucans can come from a wide variety of sources (e.g., yeast, fungi, bacteria, and some aliments); however, the macromolecular structure of β-glucans from different sources is also different and this is important to their biological functions, [167,168]. In this context, the mechanisms of action of *Saccharomyces boulardii* (a well-known yeast probiotic, also known as *Saccharomyces cerevisiae* HANSEN CBS 5926) could be indirectly link to the β-glucan-dectin-1 pathway [169,170]. However, to our knowledge, this has not yet been explored. The experiments performed by Brown et al. in 2003, using RAW264,7 cells (a mouse monocyte macrophage cell line), showed the ability of β-glucan of fungal origin to induce inflammatory mediators such as TNF-α through the action of dectin-1 and TLR-2 [171]. In line with this observation, Iliev et al. showed the role of commensal fungi (mycobiome) in the regulation of colitis via its recognition by dectin-1 [172]. Interestingly, the recent observation of Rizzetto et al. showed that chitin (another component of the fungal cell wall) from *S. cerevisiae* was able to induce trained immunity in monocytes; however, the molecular pathways involved have not been elucidated [173]. On the other hand, the association of β-glucan and probiotic bacteria (symbiotic functional foods) results in the potentiation of beneficial health effects, including immune modulation [174].

Thus, concerning polysaccharides (β-glucans and chitin) and the role of glucose metabolism in trained monocytes, a question becomes evident: What is the possibility that probiotic bacteria could use a similar pathway to stimulate the trained immunity and exert its beneficial effects? In this context, we need to keep in mind that both short chain fatty acids (SCFA, well recognized as modulators of the immune response) and lactose are the two main products of the microbial fermentation of carbohydrates. In addition, some carbohydrates, such as inulin and fructo-oligosaccharides (FOS), are considered as prebiotics and are linked to the beneficial effect of *Bifidobacterium* strains [175]. Goh and Klaenhammer, in an interesting review published in 2015, described some of the mechanisms involved in the catabolism of carbohydrates by *Lactobacillus* and *Bifidobacterium,* the most popular bacterial genera used as probiotics [176]. In addition, LAB can also synthesize exopolysaccharides (EPS) [177]. More importantly, EPS from some probiotics have been linked to anti-inflammatory and anti-cancer effects [112]. In this context, a recent review article describe the EPS produced by probiotic bacteria and cite some of their known biological properties [178]. Interestingly, Ciszek-Lendaand et al. conducted, in 2011, a study to compare the effects of Lipopolysaccharide (LPS) and EPS derived from *L. rhamnosus KL37* on the induction of pro-inflammatory cytokines (TNF-α, IL-6, and IL-12) and anti-inflammatory cytokines (IL-10) [179]. Even if both induce a pro-inflammatory profile, LPS induction was stronger and the balance of the pro- and anti-inflammatory profiles was different. They also found a relationship between the concentration of EPS used and the magnitude of the stimulation observed. Furthermore, priming with LPS reduces the inflammatory response to LPS and/or EPS re-stimulation (tolerance effect), whereas priming with EPS does not alter the re-stimulation response with LPS [179]. These observations support the idea of a putative trained immunity mechanism involving EPS that would be worth exploring in depth.

These articles, as well as many others that were not included here, suggest that innate immunity memory can be associated with the effects reported for different probiotic microorganisms; however, future investigations are necessary to understand with greater details the importance of this mechanism in the described effects.

**Figure 6:**
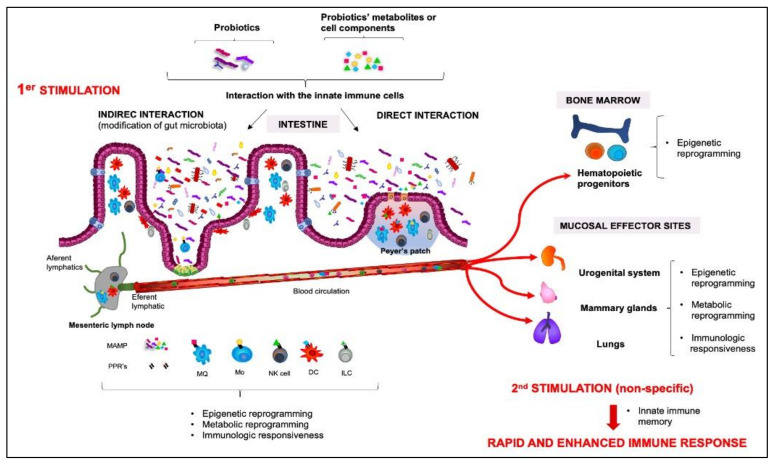
Representative model of the innate immune memory response stimulated by probiotics.

Our hypothesis is outlined in Figure 6. In addition to being able to exert beneficial changes at the level of the microbiota, and thus, stimulate trained immunity, probiotics, their metabolites, or cellular components can bind to PRRs in innate immune cells such as monocytes/macrophages, DCs and NK cells present in Peyer’s patches, or in the lamina propria. Probiotic bacteria can also be carried to the mesenteric lymph nodes (MLN) by intestinal DCs and influence the immune environment. The interaction with the immune cells induces their training, which consists of epigenetic and metabolic reprogramming. This response is also accompanied by a controlled release of cytokines (pro- or anti-inflammatory, depending on the probiotic strain) that also participates in this training. This non-specific stimulation exerted by probiotics would be related to its protective effects in different mucosal sites. Innate “memory” cells can act in the intestine, but can also reach other distant mucosal sites and respond rapidly with an enhanced immune response to secondary stimulation through the common mucosal system. Furthermore, the microbial components can travel to the bone marrow, where they can interact with pluripotent hematopoietic cells to induce long-term memory and enhance myelopoiesis, which would explain the probiotic-associated benefits observed systematically.

## 6. Conclusions and Perspectives

In conclusion, the generation of innate memory is an effective process to enhance the host’s defenses. Probiotics, by themselves or by their influence at the level of the intestinal microbiota, can participate in this process by exerting modulating effects on the signaling pathways involved in the activation and regulation/suppression of the immune response via epigenetic modifications and metabolic reprogramming. Although the term ‘trained immunity’ is not described in many studies, published works clearly show that probiotics, their components, or metabolites (postbiotics) can induce an effective innate immune memory, which would be associated with many of the benefits described both at the mucosal and systemic level. Thus, the selection of probiotic strains or the combination of probiotics, if carefully considered in the context of immune cell signaling, can achieve the desired immunomodulatory effects to boost host defense.

More studies are needed to elucidate the possible mechanisms involved in probiotic-stimulated trained immunity, and the development and standardization of these models represents a major challenge. Understanding the connection between the host, and the epigenetic and metabolic impact of probiotics, is important to identify the potential of different strains in order to select or combine them in the most convenient way to achieve the desired effect.

## Figures and Tables

**Table 1:** Trained immunity hallmarks (non-exhaustive list).

Hallmarks	Factor Measured	Trained Immunity	References
Immunologic	IL-6	Induction	[130,132]
	TNF-α	Induction	[130,132]
	PPARG (NR1C3)	Maintained	[133]
Metabolic	Glucose	Consummation increase/aerobic glycolysis	[132]
	Lactate	Production *	[132]
	Oxygen	Reduction of consummation	[132]
	mTOR	Phosphorylation/activation	[132]
	NAD+/NADH ratio	Enhanced	[132]
	Akt	Phosphorylation/activation	[132]
	p38 kinase	Phosphorylation/activation	[12]
	Raf-1 pathway	Activation	[12]
Epigenetic	H3K4	Methylation enhanced	[12,132]
	H3K27	Acetylation modulation	[132]
	Sirtuin-1	Inhibition	[132]
	HIF1	Enhanced	[132]

Abbreviations: Sirtuin-1. Histone deacetylases (HDAC) NAD+-dependent [137]. mTOR. Mammalian target of rapamycin, conserved serine/threonine-protein kinase [138]. Akt. Protein kinase. The family includes AKT1, AKT2, and AKT3. H3K4. Indicates the 4th lysine (K) residue of Histone 3 (H3). H3K27. Refers to the lysine (K) residue at the 27th position of the H3. HIF1. Hypoxia-inducible factor-1α; a transcription factor associated to carbohydrates metabolism and cancer pathways among others [139]. p38 kinase. Proline-directed serine/threonine kinases of the mitogen-activated protein kinase (MAPK) family. Known as stress-activated protein kinases [140]. Raf-1. Serine/threonine kinases involved in the MAPK cascade [141]. PPARG. Peroxisome proliferator-activated receptor gamma. Nuclear receptor which plays an important role in colon homeostasis [142]. * Related to probiotics. Note. This table was completed according to the published results cited above.

## Data Availability

Not applicable.

## References

[1] Gasteiger G., D’Osualdo A., Schubert D.A., Weber A., Bruscia E.M., Hartl D. (2017). Cellular Innate Immunity: An Old Game with New Players. J. Innate Immun..

[2] Netea M.G., Quintin J., van der Meer J.W. (2011). Trained immunity: A memory for innate host defense. Cell Host Microbe.

[3] Horvath P., Barrangou R. (2010). CRISPR/Cas, the immune system of bacteria and archaea. Science.

[4] Sorek R., Lawrence C.M., Wiedenheft B. (2013). CRISPR-mediated adaptive immune systems in bacteria and archaea. Annu. Rev. Biochem..

[5] Koonin E.V., Makarova K.S., Zhang F. (2017). Diversity, classification and evolution of CRISPR-Cas systems. Curr. Opin. Microbiol..

[6] Reimer-Michalski E.M., Conrath U. (2016). Innate immune memory in plants. Semin. Immunol..

[7] Weinhold B. (2006). Epigenetics: The science of change. Environ. Health Perspect..

[8] Handy D.E., Castro R., Loscalzo J. (2011). Epigenetic modifications: Basic mechanisms and role in cardiovascular disease. Circulation.

[9] Netea M.G., Joosten L.A.B., van der Meer J.W.M. (2017). Hypothesis: Stimulation of trained immunity as adjunctive immunotherapy in cancer. J. Leukoc. Biol..

[10] Netea M.G., Dominguez-Andres J., Barreiro L.B., Chavakis T., Divangahi M., Fuchs E., Joosten L.A.B., van der Meer J.W.M., Mhlanga M.M., Mulder W.J.M. (2020). Defining trained immunity and its role in health and disease. Nat. Rev. Immunol..

[11] Netea M.G., van der Meer J.W. (2017). Trained Immunity: An Ancient Way of Remembering. Cell Host Microbe.

[12] Quintin J., Saeed S., Martens J.H.A., Giamarellos-Bourboulis E.J., Ifrim D.C., Logie C., Jacobs L., Jansen T., Kullberg B.J., Wijmenga C. (2012). Candida albicans infection affords protection against reinfection via functional reprogramming of monocytes. Cell Host Microbe.

[13] Zhang Q., Cao X. (2019). Epigenetic regulation of the innate immune response to infection. Nat. Rev. Immunol..

[14] Jia G., Fu Y., Zhao X., Dai Q., Zheng G., Yang Y., Yi C., Lindahl T., Pan T., Yang Y.G. (2011). N6-methyladenosine in nuclear RNA is a major substrate of the obesity-associated FTO. Nat. Chem. Biol..

[15] Liu L., Song B., Ma J., Song Y., Zhang S.Y., Tang Y., Wu X., Wei Z., Chen K., Su J. (2020). Bioinformatics approaches for deciphering the epitranscriptome: Recent progress and emerging topics. Comput. Struct. Biotechnol. J..

[16] Burgess A., David R., Searle I.R. (2016). Deciphering the epitranscriptome: A green perspective. J. Integr. Plant Biol..

[17] Kleinnijenhuis J., Quintin J., Preijers F., Joosten L.A., Ifrim D.C., Saeed S., Jacobs C., van Loenhout J., de Jong D., Stunnenberg H.G. (2012). Bacille Calmette-Guerin induces NOD2-dependent nonspecific protection from reinfection via epigenetic reprogramming of monocytes. Proc. Natl. Acad. Sci. USA.

[18] DiNardo A.R., Netea M.G., Musher D.M. (2021). Postinfectious Epigenetic Immune Modifications - A Double-Edged Sword. N. Engl. J. Med..

[19] Khader S.A., Divangahi M., Hanekom W., Hill P.C., Maeurer M., Makar K.W., Mayer-Barber K.D., Mhlanga M.M., Nemes E., Schlesinger L.S. (2019). Targeting innate immunity for tuberculosis vaccination. J. Clin. Investig..

[20] Koeken V., van Crevel R., Netea M.G., Li Y. (2021). Resolving trained immunity with systems biology. Eur. J. Immunol..

[21] Lyko F. (2018). The DNA methyltransferase family: A versatile toolkit for epigenetic regulation. Nat. Rev. Genet..

[22] Hauser A.T., Robaa D., Jung M. (2018). Epigenetic small molecule modulators of histone and DNA methylation. Curr. Opin. Chem. Biol..

[23] Rice J.C., Allis C.D. (2001). Histone methylation versus histone acetylation: New insights into epigenetic regulation. Curr. Opin. Cell Biol..

[24] Grunstein M. (1997). Histone acetylation in chromatin structure and transcription. Nature.

[25] Biel M., Wascholowski V., Giannis A. (2005). Epigenetics—An epicenter of gene regulation: Histones and histone-modifying enzymes. Angew. Chem. Int. Ed. Engl..

[26] Perillo B., Tramontano A., Pezone A., Migliaccio A. (2020). LSD1: More than demethylation of histone lysine residues. Exp. Mol. Med..

[27] Combes G., Alharbi I., Braga L.G., Elowe S. (2017). Playing polo during mitosis: PLK1 takes the lead. Oncogene.

[28] Davie J.R., Spencer V.A. (1999). Control of histone modifications. J. Cell Biochem..

[29] Clapier C.R., Iwasa J., Cairns B.R., Peterson C.L. (2017). Mechanisms of action and regulation of ATP-dependent chromatin-remodelling complexes. Nat. Rev. Mol. Cell Biol..

[30] Becker P.B., Horz W. (2002). ATP-dependent nucleosome remodeling. Annu. Rev. Biochem..

[31] Esteller M. (2011). Non-coding RNAs in human disease. Nat. Rev. Genet..

[32] Sen R., Ghosal S., Das S., Balti S., Chakrabarti J. (2014). Competing endogenous RNA: The key to posttranscriptional regulation. Sci. World J..

[33] Chen X., Yang T., Wang W., Xi W., Zhang T., Li Q., Yang A., Wang T. (2019). Circular RNAs in immune responses and immune diseases. Theranostics.

[34] Sartorelli V., Lauberth S.M. (2020). Enhancer RNAs are an important regulatory layer of the epigenome. Nat. Struct. Mol. Biol..

[35] Meng H., Bartholomew B. (2018). Emerging roles of transcriptional enhancers in chromatin looping and promoter-proximal pausing of RNA polymerase II. J. Biol. Chem..

[36] Esteve-Puig R., Bueno-Costa A., Esteller M. (2020). Writers, readers and erasers of RNA modifications in cancer. Cancer Lett..

[37] Nie F., Feng P., Song X., Wu M., Tang Q., Chen W. (2020). RNAWRE: A resource of writers, readers and erasers of RNA modifications. Database.

[38] Shenderov B.A. (2012). Gut indigenous microbiota and epigenetics. Microb. Ecol. Health Dis..

[39] Carbonero F. (2017). Human epigenetics and microbiome: The potential for a revolution in both research areas by integrative studies. Future Sci. OA.

[40] Nackerdien Z.E. (2008). Perspectives on microbes as oncogenic infectious agents and implications for breast cancer. Med. Hypotheses.

[41] Licciardi P.V., Wong S.S., Tang M.L., Karagiannis T.C. (2010). Epigenome targeting by probiotic metabolites. Gut Pathog..

[42] Canani R.B., Costanzo M.D., Leone L., Bedogni G., Brambilla P., Cianfarani S., Nobili V., Pietrobelli A., Agostoni C. (2011). Epigenetic mechanisms elicited by nutrition in early life. Nutr. Res. Rev..

[43] Worthley D.L., Le Leu R.K., Whitehall V.L., Conlon M., Christophersen C., Belobrajdic D., Mallitt K.A., Hu Y., Irahara N., Ogino S. (2009). A human, double-blind, placebo-controlled, crossover trial of prebiotic, probiotic, and synbiotic supplementation: Effects on luminal, inflammatory, epigenetic, and epithelial biomarkers of colorectal cancer. Am. J. Clin. Nutr..

[44] Rosenberg E., Zilber-Rosenberg I. (2011). Symbiosis and development: The hologenome concept. Birth Defects Res. C Embryo Today.

[45] Azad M.B., Kozyrskyj A.L. (2012). Perinatal programming of asthma: The role of gut microbiota. Clin. Dev. Immunol..

[46] West C.E., D’Vaz N., Prescott S.L. (2011). Dietary immunomodulatory factors in the development of immune tolerance. Curr. Allergy Asthma Rep..

[47] Bebek G., Bennett K.L., Funchain P., Campbell R., Seth R., Scharpf J., Burkey B., Eng C. (2012). Microbiomic subprofiles and MDR1 promoter methylation in head and neck squamous cell carcinoma. Hum. Mol. Genet..

[48] Hamon M.A., Cossart P. (2008). Histone modifications and chromatin remodeling during bacterial infections. Cell Host Microbe.

[49] Candelli M., Franza L., Pignataro G., Ojetti V., Covino M., Piccioni A., Gasbarrini A., Franceschi F. (2021). Interaction between Lipopolysaccharide and Gut Microbiota in Inflammatory Bowel Diseases. Int. J. Mol. Sci..

[50] Ghadimi D., Helwig U., Schrezenmeir J., Heller K.J., de Vrese M. (2012). Epigenetic imprinting by commensal probiotics inhibits the IL-23/IL-17 axis in an in vitro model of the intestinal mucosal immune system. J. Leukoc. Biol..

[51] Kumar M., Nagpal R., Verma V., Kumar A., Kaur N., Hemalatha R., Gautam S.K., Singh B. (2013). Probiotic metabolites as epigenetic targets in the prevention of colon cancer. Nutr. Rev..

[52] Panwar H., Rashmi H.M., Batish V.K., Grover S. (2013). Probiotics as potential biotherapeutics in the management of type 2 diabetes - prospects and perspectives. Diabetes Metab. Res. Rev..

[53] Sestito S., D’Auria E., Baldassarre M.E., Salvatore S., Tallarico V., Stefanelli E., Tarsitano F., Concolino D., Pensabene L. (2020). The Role of Prebiotics and Probiotics in Prevention of Allergic Diseases in Infants. Front. Pediatr..

[54] Bhat M.I., Kapila R. (2017). Dietary metabolites derived from gut microbiota: Critical modulators of epigenetic changes in mammals. Nutr. Rev..

[55] Lenoir M., Martín R., Torres-Maravilla E., Chadi S., González-Dávila P., Sokol H., Langella P., Chain F., Bermúdez-Humarán L.G. (2020). Butyrate mediates anti-inflammatory effects of Faecalibacterium prausnitzii in intestinal epithelial cells through Dact3. Gut Microbes.

[56] Amarante-Mendes G.P., Adjemian S., Branco L.M., Zanetti L.C., Weinlich R., Bortoluci K.R. (2018). Pattern Recognition Receptors and the Host Cell Death Molecular Machinery. Front. Immunol..

[57] Medzhitov R., Janeway C.A. (2002). Decoding the patterns of self and nonself by the innate immune system. Science.

[58] Koenderman L., Buurman W., Daha M.R. (2014). The innate immune response. Immunol. Lett..

[59] Canton J., Neculai D., Grinstein S. (2013). Scavenger receptors in homeostasis and immunity. Nat. Rev. Immunol..

[60] Aderem A., Ulevitch R.J. (2000). Toll-like receptors in the induction of the innate immune response. Nature.

[61] Matzinger P. (2002). The danger model: A renewed sense of self. Science.

[62] Zindel J., Kubes P. (2020). DAMPs, PAMPs, and LAMPs in Immunity and Sterile Inflammation. Annu. Rev. Pathol..

[63] PrabhuDas M.R., Baldwin C.L., Bollyky P.L., Bowdish D.M.E., Drickamer K., Febbraio M., Herz J., Kobzik L., Krieger M., Loike J. (2017). A Consensus Definitive Classification of Scavenger Receptors and Their Roles in Health and Disease. J. Immunol..

[64] Del Fresno C., Cueto F.J., Sancho D. (2019). A Proposal for Nomenclature in Myeloid C-Type Lectin Receptors. Front. Immunol..

[65] Figdor C.G., van Kooyk Y., Adema G.J. (2002). C-type lectin receptors on dendritic cells and Langerhans cells. Nat. Rev. Immunol..

[66] Liu G., Lu Y., Thulasi Raman S.N., Xu F., Wu Q., Li Z., Brownlie R., Liu Q., Zhou Y. (2018). Nuclear-resident RIG-I senses viral replication inducing antiviral immunity. Nat. Commun..

[67] Uhlén M., Fagerberg L., Hallström B.M., Lindskog C., Oksvold P., Mardinoglu A., Sivertsson Å., Kampf C., Sjöstedt E., Asplund A. (2015). Proteomics. Tissue-based map of the human proteome. Science.

[68] Pombinho R., Sousa S., Cabanes D. (2018). Scavenger Receptors: Promiscuous Players during Microbial Pathogenesis. Crit. Rev. Microbiol..

[69] Hoving J.C., Wilson G.J., Brown G.D. (2014). Signalling C-type lectin receptors, microbial recognition and immunity. Cell. Microbiol..

[70] Geijtenbeek T.B., Gringhuis S.I. (2009). Signalling through C-type lectin receptors: Shaping immune responses. Nat. Rev. Immunol..

[71] Ganguly K., Kishore U., Madan T. (2021). Interplay between C-type lectin receptors and microRNAs in cellular homeostasis and immune response. FEBS J..

[72] Brown G.D., Willment J.A., Whitehead L. (2018). C-type lectins in immunity and homeostasis. Nat. Rev. Immunol..

[73] Mokhtari Y., Pourbagheri-Sigaroodi A., Zafari P., Bagheri N., Ghaffari S.H., Bashash D. (2021). Toll-like receptors (TLRs): An old family of immune receptors with a new face in cancer pathogenesis. J. Cell. Mol. Med..

[74] Kawai T., Akira S. (2010). The role of pattern-recognition receptors in innate immunity: Update on Toll-like receptors. Nat. Immunol..

[75] Liu M., Zen K. (2021). Toll-Like Receptors Regulate the Development and Progression of Renal Diseases. Kidney Dis..

[76] Onomoto K., Onoguchi K., Yoneyama M. (2021). Regulation of RIG-I-like receptor-mediated signaling: Interaction between host and viral factors. Cell. Mol. Immunol..

[77] Rehwinkel J., Gack M.U. (2020). RIG-I-like receptors: Their regulation and roles in RNA sensing. Nat. Rev. Immunol..

[78] Kanneganti T.D., Lamkanfi M., Nunez G. (2007). Intracellular NOD-like receptors in host defense and disease. Immunity.

[79] Guo H., Gibson S.A., Ting J.P.Y. (2020). Gut microbiota, NLR proteins, and intestinal homeostasis. J. Exp. Med..

[80] Saur I.M.L., Panstruga R., Schulze-Lefert P. (2021). NOD-like receptor-mediated plant immunity: From structure to cell death. Nat. Rev. Immunol..

[81] Abrahams V.M. (2011). The role of the Nod-like receptor family in trophoblast innate immune responses. J. Reprod. Immunol..

[82] Chen Q., Sun L., Chen Z.J. (2016). Regulation and function of the cGAS-STING pathway of cytosolic DNA sensing. Nat. Immunol..

[83] Sun L., Wu J., Du F., Chen X., Chen Z.J. (2013). Cyclic GMP-AMP synthase is a cytosolic DNA sensor that activates the type I interferon pathway. Science.

[84] Wan D., Jiang W., Hao J. (2020). Research Advances in How the cGAS-STING Pathway Controls the Cellular Inflammatory Response. Front. Immunol..

[85] O’Neill L.A., Kishton R.J., Rathmell J. (2016). A guide to immunometabolism for immunologists. Nat. Rev. Immunol..

[86] Baron M.H., Isern J., Fraser S.T. (2012). The embryonic origins of erythropoiesis in mammals. Blood.

[87] Cedar H., Bergman Y. (2011). Epigenetics of haematopoietic cell development. Nat. Rev. Immunol..

[88] Crisan M., Dzierzak E. (2016). The many faces of hematopoietic stem cell heterogeneity. Development.

[89] Cheng H., Zheng Z., Cheng T. (2020). New paradigms on hematopoietic stem cell differentiation. Protein Cell.

[90] Bedoui S., Gebhardt T., Gasteiger G., Kastenmüller W. (2016). Parallels and differences between innate and adaptive lymphocytes. Nat. Immunol..

[91] Psaila B., Mead A.J. (2019). Single-cell approaches reveal novel cellular pathways for megakaryocyte and erythroid differentiation. Blood.

[92] Kabelitz D. (2007). Expression and function of Toll-like receptors in T lymphocytes. Curr. Opin. Immunol..

[93] Crellin N.K., Trifari S., Kaplan C.D., Satoh-Takayama N., Di Santo J.P., Spits H. (2010). Regulation of cytokine secretion in human CD127(+) LTi-like innate lymphoid cells by Toll-like receptor 2. Immunity.

[94] Pluddemann A., Mukhopadhyay S., Gordon S. (2011). Innate immunity to intracellular pathogens: Macrophage receptors and responses to microbial entry. Immunol. Rev..

[95] Gurtner A., Gonzalez-Perez I., Arnold I.C. (2021). Intestinal eosinophils, homeostasis and response to bacterial intrusion. Semin. Immunopathol..

[96] Shamri R., Xenakis J.J., Spencer L.A. (2011). Eosinophils in innate immunity: An evolving story. Cell Tissue Res..

[97] Kvarnhammar A.M., Cardell L.O. (2012). Pattern-recognition receptors in human eosinophils. Immunology.

[98] Pellefigues C., Mehta P., Chappell S., Yumnam B., Old S., Camberis M., Le Gros G. (2021). Diverse innate stimuli activate basophils through pathways involving Syk and IkappaB kinases. Proc. Natl. Acad. Sci. USA.

[99] Qiu H.N., Wong C.K., Chu I.M., Hu S., Lam C.W. (2013). Muramyl dipeptide mediated activation of human bronchial epithelial cells interacting with basophils: A novel mechanism of airway inflammation. Clin. Exp. Immunol..

[100] Bieneman A.P., Chichester K.L., Chen Y.H., Schroeder J.T. (2005). Toll-like receptor 2 ligands activate human basophils for both IgE-dependent and IgE-independent secretion. J. Allergy Clin. Immunol..

[101] Jeon J.H., Ahn K.B., Kim S.K., Im J., Yun C.H., Han S.H. (2015). Bacterial flagellin induces IL-6 expression in human basophils. Mol. Immunol..

[102] Yousefi S., Morshed M., Amini P., Stojkov D., Simon D., von Gunten S., Kaufmann T., Simon H.U. (2015). Basophils exhibit antibacterial activity through extracellular trap formation. Allergy.

[103] Espinosa-Riquer Z.P., Segura-Villalobos D., Ramirez-Moreno I.G., Perez Rodriguez M.J., Lamas M., Gonzalez-Espinosa C. (2020). Signal Transduction Pathways Activated by Innate Immunity in Mast Cells: Translating Sensing of Changes into Specific Responses. Cells.

[104] Agier J., Pastwinska J., Brzezinska-Blaszczyk E. (2018). An overview of mast cell pattern recognition receptors. Inflamm. Res..

[105] Koller B., Bals R., Roos D., Korting H.C., Griese M., Hartl D. (2009). Innate immune receptors on neutrophils and their role in chronic lung disease. Eur. J. Clin. Investig..

[106] Mantovani A., Cassatella M.A., Costantini C., Jaillon S. (2011). Neutrophils in the activation and regulation of innate and adaptive immunity. Nat. Rev. Immunol..

[107] Rungelrath V., Kobayashi S.D., DeLeo F.R. (2020). Neutrophils in innate immunity and systems biology-level approaches. Wiley Interdiscip. Rev. Syst. Biol. Med..

[108] Dzopalic T., Rajkovic I., Dragicevic A., Colic M. (2012). The response of human dendritic cells to co-ligation of pattern-recognition receptors. Immunol. Res..

[109] Choreno-Parra J.A., Weinstein L.I., Yunis E.J., Zuniga J., Hernandez-Pando R. (2020). Thinking Outside the Box: Innate- and B Cell-Memory Responses as Novel Protective Mechanisms Against Tuberculosis. Front. Immunol..

[110] Adib-Conquy M., Scott-Algara D., Cavaillon J.M., Souza-Fonseca-Guimaraes F. (2014). TLR-mediated activation of NK cells and their role in bacterial/viral immune responses in mammals. Immunol. Cell Biol..

[111] Stokic-Trtica V., Diefenbach A., Klose C.S.N. (2020). NK Cell Development in Times of Innate Lymphoid Cell Diversity. Front. Immunol..

[112] Wang L., Wang Y., Li Q., Tian K., Xu L., Liu G., Guo C. (2019). Exopolysaccharide, Isolated From a Novel Strain Bifidobacterium breve lw01 Possess an Anticancer Effect on Head and Neck Cancer—Genetic and Biochemical Evidences. Front. Microbiol..

[113] Martinez-Gonzalez I., Ghaedi M., Steer C.A., Matha L., Vivier E., Takei F. (2018). ILC2 memory: Recollection of previous activation. Immunol. Rev..

[114] Eberl G., Colonna M., Di Santo J.P., McKenzie A.N. (2015). Innate lymphoid cells. Innate lymphoid cells: A new paradigm in immunology. Science.

[115] Pavlovic M., Gross C., Chili C., Secher T., Treiner E. (2020). MAIT Cells Display a Specific Response to Type 1 IFN Underlying the Adjuvant Effect of TLR7/8 Ligands. Front. Immunol..

[116] Legoux F., Salou M., Lantz O. (2020). MAIT Cell Development and Functions: The Microbial Connection. Immunity.

[117] Dias J., Leeansyah E., Sandberg J.K. (2017). Multiple layers of heterogeneity and subset diversity in human MAIT cell responses to distinct microorganisms and to innate cytokines. Proc. Natl. Acad. Sci. USA.

[118] Provine N.M., Klenerman P. (2020). MAIT Cells in Health and Disease. Annu. Rev. Immunol..

[119] Mattner J., Debord K.L., Ismail N., Goff R.D., Cantu C., Zhou D., Saint-Mezard P., Wang V., Gao Y., Yin N. (2005). Exogenous and endogenous glycolipid antigens activate NKT cells during microbial infections. Nature.

[120] Van Kaer L., Parekh V.V., Wu L. (2015). The Response of CD1d-Restricted Invariant NKT Cells to Microbial Pathogens and Their Products. Front. Immunol..

[121] Kronenberg M., Kinjo Y. (2009). Innate-like recognition of microbes by invariant natural killer T cells. Curr. Opin. Immunol..

[122] Brennan P.J., Brigl M., Brenner M.B. (2013). Invariant natural killer T cells: An innate activation scheme linked to diverse effector functions. Nat. Rev. Immunol..

[123] Olszak T., An D., Zeissig S., Vera M.P., Richter J., Franke A., Glickman J.N., Siebert R., Baron R.M., Kasper D.L. (2012). Microbial exposure during early life has persistent effects on natural killer T cell function. Science.

[124] Cheroutre H., Lambolez F., Mucida D. (2011). The light and dark sides of intestinal intraepithelial lymphocytes. Nat. Rev. Immunol..

[125] Hayday A., Theodoridis E., Ramsburg E., Shires J. (2001). Intraepithelial lymphocytes: Exploring the Third Way in immunology. Nat. Immunol..

[126] Jiang W., Wang X., Zeng B., Liu L., Tardivel A., Wei H., Han J., MacDonald H.R., Tschopp J., Tian Z. (2013). Recognition of gut microbiota by NOD2 is essential for the homeostasis of intestinal intraepithelial lymphocytes. J. Exp. Med..

[127] Lepage A.C., Buzoni-Gatel D., Bout D.T., Kasper L.H. (1998). Gut-derived intraepithelial lymphocytes induce long term immunity against Toxoplasma gondii. J. Immunol..

[128] Wesch D., Beetz S., Oberg H.H., Marget M., Krengel K., Kabelitz D. (2006). Direct costimulatory effect of TLR3 ligand poly(I:C) on human gamma delta T lymphocytes. J. Immunol..

[129] Divangahi M., Aaby P., Khader S.A., Barreiro L.B., Bekkering S., Chavakis T., van Crevel R., Curtis N., DiNardo A.R., Dominguez-Andres J. (2021). Trained immunity, tolerance, priming and differentiation: Distinct immunological processes. Nat. Immunol..

[130] Ifrim D.C., Quintin J., Joosten L.A., Jacobs C., Jansen T., Jacobs L., Gow N.A., Williams D.L., van der Meer J.W., Netea M.G. (2014). Trained immunity or tolerance: Opposing functional programs induced in human monocytes after engagement of various pattern recognition receptors. Clin. Vaccine Immunol..

[131] Eljaszewicz A., Ruchti F., Radzikowska U., Globinska A., Boonpiyathad T., Gschwend A., Morita H., Helbling A., Arasi S., Kahlert H. (2021). Trained immunity and tolerance in innate lymphoid cells, monocytes, and dendritic cells during allergen-specific immunotherapy. J. Allergy Clin. Immunol..

[132] Cheng S.C., Quintin J., Cramer R.A., Shepardson K.M., Saeed S., Kumar V., Giamarellos-Bourboulis E.J., Martens J.H., Rao N.A., Aghajanirefah A. (2014). mTOR- and HIF-1alpha-mediated aerobic glycolysis as metabolic basis for trained immunity. Science.

[133] Saeed S., Quintin J., Kerstens H.H., Rao N.A., Aghajanirefah A., Matarese F., Cheng S.C., Ratter J., Berentsen K., van der Ent M.A. (2014). Epigenetic programming of monocyte-to-macrophage differentiation and trained innate immunity. Science.

[134] Larabee J.L., Shakir S.M., Barua S., Ballard J.D. (2013). Increased cAMP in monocytes augments Notch signaling mechanisms by elevating RBP-J and transducin-like enhancer of Split (TLE). J. Biol. Chem..

[135] Serezani C.H., Ballinger M.N., Aronoff D.M., Peters-Golden M. (2008). Cyclic AMP: Master regulator of innate immune cell function. Am. J. Respir. Cell Mol. Biol..

[136] Biswas S.K., Lopez-Collazo E. (2009). Endotoxin tolerance: New mechanisms, molecules and clinical significance. Trends Immunol..

[137] Perez V., Tellechea J., Corpa J.M., Gutierrez M., Garcia Marin J.F. (1999). Relation between pathologic findings and cellular immune responses in sheep with naturally acquired paratuberculosis. Am. J. Vet. Res..

[138] Danesh Pazhooh R., Rahnamay Farnood P., Asemi Z., Mirsafaei L., Yousefi B., Mirzaei H. (2021). mTOR pathway and DNA damage response: A therapeutic strategy in cancer therapy. DNA Repair.

[139] Slemc L., Kunej T. (2016). Transcription factor HIF1A: Downstream targets, associated pathways, polymorphic hypoxia response element (HRE) sites, and initiative for standardization of reporting in scientific literature. Tumour Biol..

[140] Canovas B., Nebreda A.R. (2021). Diversity and versatility of p38 kinase signalling in health and disease. Nat. Rev. Mol. Cell Biol..

[141] Roskoski R. (2010). RAF protein-serine/threonine kinases: Structure and regulation. Biochem. Biophys. Res. Commun..

[142] Peyrin-Biroulet L., Beisner J., Wang G., Nuding S., Oommen S.T., Kelly D., Parmentier-Decrucq E., Dessein R., Merour E., Chavatte P. (2010). Peroxisome proliferator-activated receptor gamma activation is required for maintenance of innate antimicrobial immunity in the colon. Proc. Natl. Acad. Sci. USA.

[143] Dudek-Wicher R., Junka A., Paleczny J., Bartoszewicz M. (2020). Clinical Trials of Probiotic Strains in Selected Disease Entities. Int. J. Microbiol..

[144] Maldonado Galdeano C., Cazorla S.I., Lemme Dumit J.M., Vélez E., Perdigón G. (2019). Beneficial Effects of Probiotic Consumption on the Immune System. Ann. Nutr. Metab..

[145] Khalesi S., Bellissimo N., Vandelanotte C., Williams S., Stanley D., Irwin C. (2019). A review of probiotic supplementation in healthy adults: Helpful or hype?. Eur. J. Clin. Nutr..

[146] Torpee S., Kantachote D., Rattanachuay P., Chiayvareesajja S., Tantirungkij M. (2021). Dietary supplementation with probiotic Rhodobacter sphaeroides SS15 extract to control acute hepatopancreatic necrosis disease (AHPND)-causing Vibrio parahaemolyticus in cultivated white shrimp. J. Invertebr. Pathol..

[147] de Moreno de LeBlanc A., LeBlanc J.G. (2014). Effect of probiotic administration on the intestinal microbiota, current knowledge and potential applications. World J. Gastroenterol..

[148] Negi S., Das D.K., Pahari S., Nadeem S., Agrewala J.N. (2019). Potential Role of Gut Microbiota in Induction and Regulation of Innate Immune Memory. Front. Immunol..

[149] Belkaid Y., Harrison O.J. (2017). Homeostatic Immunity and the Microbiota. Immunity.

[150] Mileti E., Matteoli G., Iliev I.D., Rescigno M. (2009). Comparison of the immunomodulatory properties of three probiotic strains of Lactobacilli using complex culture systems: Prediction for in vivo efficacy. PLoS ONE.

[151] Llewellyn A., Foey A. (2017). Probiotic Modulation of Innate Cell Pathogen Sensing and Signaling Events. Nutrients.

[152] Castillo N.A., Perdigon G., de Moreno de Leblanc A. (2011). Oral administration of a probiotic Lactobacillus modulates cytokine production and TLR expression improving the immune response against Salmonella enterica serovar Typhimurium infection in mice. BMC Microbiol..

[153] Villena J., Vizoso-Pinto M.G., Kitazawa H. (2016). Intestinal Innate Antiviral Immunity and Immunobiotics: Beneficial Effects against Rotavirus Infection. Front. Immunol..

[154] Ganguli K., Meng D., Rautava S., Lu L., Walker W.A., Nanthakumar N. (2013). Probiotics prevent necrotizing enterocolitis by modulating enterocyte genes that regulate innate immune-mediated inflammation. Am. J. Physiol. Gastrointest. Liver Physiol..

[155] Sham H.P., Yu E.Y., Gulen M.F., Bhinder G., Stahl M., Chan J.M., Brewster L., Morampudi V., Gibson D.L., Hughes M.R. (2013). SIGIRR, a negative regulator of TLR/IL-1R signalling promotes Microbiota dependent resistance to colonization by enteric bacterial pathogens. PLoS Pathog..

[156] Salva S., Alvarez S. (2017). The Role of Microbiota and Immunobiotics in Granulopoiesis of Immunocompromised Hosts. Front. Immunol..

[157] Salva S., Marranzino G., Villena J., Agüero G., Alvarez S. (2014). Probiotic Lactobacillus strains protect against myelosuppression and immunosuppression in cyclophosphamide-treated mice. Int. Immunopharmacol..

[158] Herrera M., Salva S., Villena J., Barbieri N., Marranzino G., Alvarez S. (2014). Dietary supplementation with Lactobacilli improves emergency granulopoiesis in protein-malnourished mice and enhances respiratory innate immune response. PLoS ONE.

[159] de Moreno de LeBlanc A., Dogi C.A., Galdeano C.M., Carmuega E., Weill R., Perdigon G. (2008). Effect of the administration of a fermented milk containing Lactobacillus casei DN-114001 on intestinal microbiota and gut associated immune cells of nursing mice and after weaning until immune maturity. BMC Immunol..

[160] de Moreno de LeBlanc A., Maldonado Galdeano C., Dogi C.A., Carmuega E., Weill R., Perdigon G. (2010). Adjuvant effect of a probiotic fermented milk in the protection against Salmonella enteritidis serovar typhimurium infection: Mechanisms involved. Int. J. Immunopathol. Pharmacol..

[161] Mortaz E., Adcock I.M., Folkerts G., Barnes P.J., Paul Vos A., Garssen J. (2013). Probiotics in the management of lung diseases. Mediat. inflamm..

[162] Kawahara T., Takahashi T., Oishi K., Tanaka H., Masuda M., Takahashi S., Takano M., Kawakami T., Fukushima K., Kanazawa H. (2015). Consecutive oral administration of Bifidobacterium longum MM-2 improves the defense system against influenza virus infection by enhancing natural killer cell activity in a murine model. Microbiol. Immunol..

[163] Garcia-Castillo V., Tomokiyo M., Raya Tonetti F., Islam M.A., Takahashi H., Kitazawa H., Villena J. (2020). Alveolar Macrophages Are Key Players in the Modulation of the Respiratory Antiviral Immunity Induced by Orally Administered Lacticaseibacillus rhamnosus CRL1505. Front. Immunol..

[164] Koizumi S., Wakita D., Sato T., Mitamura R., Izumo T., Shibata H., Kiso Y., Chamoto K., Togashi Y., Kitamura H. (2008). Essential role of Toll-like receptors for dendritic cell and NK1.1(+) cell-dependent activation of type 1 immunity by Lactobacillus pentosus strain S-PT84. Immunol. Lett..

[165] Forsythe P. (2014). Probiotics and lung immune responses. Ann. Am. Thorac. Soc..

[166] Dong H., Rowland I., Yaqoob P. (2012). Comparative effects of six probiotic strains on immune function in vitro. Br. J. Nutr..

[167] Du B., Meenu M., Liu H., Xu B. (2019). A Concise Review on the Molecular Structure and Function Relationship of beta-Glucan. Int. J. Mol. Sci..

[168] Goodridge H.S., Wolf A.J., Underhill D.M. (2009). Beta-glucan recognition by the innate immune system. Immunol. Rev..

[169] Stier H., Bischoff S.C. (2016). Influence of Saccharomyces boulardii CNCM I-745on the gut-associated immune system. Clin. Exp. Gastroenterol..

[170] Sen S., Mansell T.J. (2020). Yeasts as probiotics: Mechanisms, outcomes, and future potential. Fungal Genet. Biol..

[171] Brown G.D., Herre J., Williams D.L., Willment J.A., Marshall A.S., Gordon S. (2003). Dectin-1 mediates the biological effects of beta-glucans. J. Exp. Med..

[172] Iliev I.D., Funari V.A., Taylor K.D., Nguyen Q., Reyes C.N., Strom S.P., Brown J., Becker C.A., Fleshner P.R., Dubinsky M. (2012). Interactions between commensal fungi and the C-type lectin receptor Dectin-1 influence colitis. Science.

[173] Rizzetto L., Ifrim D.C., Moretti S., Tocci N., Cheng S.C., Quintin J., Renga G., Oikonomou V., De Filippo C., Weil T. (2016). Fungal Chitin Induces Trained Immunity in Human Monocytes during Cross-talk of the Host with Saccharomyces cerevisiae. J. Biol. Chem..

[174] Mattia Pia A., Giuseppe S., Daniela F. (2017). β-Glucans and Probiotics. Am. J. Immunol..

[175] Salazar N., Ruas-Madiedo P., Kolida S., Collins M., Rastall R., Gibson G., de Los Reyes-Gavilán C.G. (2009). Exopolysaccharides produced by Bifidobacterium longum IPLA E44 and Bifidobacterium animalis subsp. lactis IPLA R1 modify the composition and metabolic activity of human faecal microbiota in pH-controlled batch cultures. Int. J. Food Microbiol..

[176] Goh Y.J., Klaenhammer T.R. (2015). Genetic mechanisms of prebiotic oligosaccharide metabolism in probiotic microbes. Annu. Rev. Food Sci. Technol..

[177] Zhang M., Lai T., Yao M., Zhang M., Yang Z. (2021). Interaction of the Exopolysaccharide from Lactobacillus plantarum YW11 with Casein and Bioactivities of the Polymer Complex. Foods.

[178] Angelin J., Kavitha M. (2020). Exopolysaccharides from probiotic bacteria and their health potential. Int. J. Biol. Macromol..

[179] Ciszek-Lenda M., Nowak B., Srottek M., Gamian A., Marcinkiewicz J. (2011). Immunoregulatory potential of exopolysaccharide from Lactobacillus rhamnosus KL37: Effects on the production of inflammatory mediators by mouse macrophages. Int. J. Exp. Pathol..

